# Experimental and data-driven analysis for predicting nanofluid performance in improving foam stability and reducing mobility at critical micelle concentration

**DOI:** 10.1038/s41598-024-58609-3

**Published:** 2024-04-03

**Authors:** Miras Issakhov, Maral Khanjani, Adiya Muratkhozhina, Peyman Pourafshary, Saule Aidarova, Altynay Sharipova

**Affiliations:** 1 School of Mining and Geosciences, Nazarbayev University, Astana, Kazakhstan; 2 Kazakh-British Technical University, Almaty, Kazakhstan; 3 Satbayev University, Almaty, Kazakhstan

**Keywords:** Engineering, Nanoscience and technology

## Abstract

Application of surfactant-based foam flooding is an effective approach to reduce mobility and control early breakthrough. Despite the proper performance of surfactant-based foams in decreasing the channeling of the flooded gas and water, high pressure, high temperature, and high salinity of the reservoirs put some limitations on the foam flooding efficiency. Nanoparticles are used to improve the quality of the foams, enhance stability, and transcend the limitations. Although there are many benefits of using nanoparticles in foam flooding, their performance at surfactant critical micelle concentration (CMC) is not fully investigated and the optimum nanoparticle concentration is not specified. In this study, an experimental investigation using nanosilica with surfactants at CMC to improve the stability (half-life) and mobility reduction factor (MRF) has been conducted. Furthermore, data from the literature were collected and analyzed to evaluate the change in MRF and stability for a nanofluid-based foam at CMC. Both experimental results and literature data showed that application of nanofluid-based foam is a successful approach to develop a more stable foam with lower mobility. Nanoparticle (NP) concentration is the dominant parameter at different salinities and temperatures that affects foam flow through porous media. The range of 0.2–0.4 wt% is the optimum nanoparticle concentration to develop a strong foam with acceptable performance in controlling mobility.

## Introduction

There are different challenges in the petroleum industry regarding producing oil from a reservoir. EOR (enhanced oil recovery) methods became popular due to the depletion of the pressure in reservoirs^1–3^. Gas flooding is one of the most used EOR methods, which is more efficient in comparison to the other methods^3–8^. Despite many advantages, gas flooding has challenges^4^. Firstly, the high difference between the density of the injected gas and the underground fluid causes issues such as gravity override, in which the injected gas migrates to the top section and the oil might remain unswept^9,10^. Secondly, gas flooding has a very low sweep efficiency due to high mobility and low viscosity, which causes viscous fingering and early breakthrough^11–14^. Moreover, the heterogeneity in reservoirs causes flow channeling and low vertical sweep efficiency^15,16^. In order to mitigate the above-mentioned issues and enhance the control of the mobility of the injected gas, surfactants are added for foam generation.^17–27^. The main purpose of surfactants is to reduce the interfacial tension (IFT) between residual oil and injected gad and increase the gas viscosity, as well as density, which improves the oil recovery^9,17^. Different kinds of surfactants are classified into four groups of anionic, cationic, amphoteric, and nonionic. Among these, anionic surfactants such as α-olefin sulfonate (AOS) and sodium dodecyl sulfate (SDS) are the most used materials for generating efficient foams^23^.

However, harsh conditions in the reservoirs such as high pressure, high temperature, and high salinity might have a negative impact on surfactant-based foam efficiency^28,29^. Surfactants in these situations are prone to be more adsorbed to the rock surface and might lose their ability to maintain the foam stability^24^. Hence, different approaches are examined to improve the quality and stability of foams. Over the last few years, the application of nanoparticles has been widely studied in order to enhance the surfactant-based foam flooding performance in the reservoirs^21,22^ since nanoparticles reduce the surface tension between the liquid and gas phases in the foam. Moreover, they cover like a shield over the surfactant particles, which leads to strengthening the foam bubbles. In addition, nanoparticles decrease the surfactant adsorption on the rock, which might increase the foam stability and delay the foam drainage^21,22^. Furthermore, nanoparticles play a key role in increasing the viscosity of the foams. Due to the high viscosity of the oil in the reservoirs, an increase in nanofluid-assisted foam viscosity affects the mobility ratio and improves the oil recovery.

The stability of foams can be evaluated by static tests such as foamability and measurement of the change in the height of the foam over time^28^. There are two main parameters assessed by the above-mentioned tests. First one is half-life which is the time required for the foam volume or height to decrease to its half. Another parameter considered in the foam flooding is mobility reduction factor (MRF). MRF is the alteration in the gas mobility which is indicated by the change in pressure drop along a porous medium during foam injection^30^. The efficiency of the foam evaluated by MRF can be shown in Eq. (1):

1 $$MRF=\frac{{(\frac{KA\Delta P}{QL})}_{(f)}}{{(\frac{KA\Delta P}{QL})}_{(g)}}=\frac{{\Delta P}_{f}}{{\Delta P}_{g}}$$

where K is the absolute permeability, A is the cross-section area of the core, Q is the flow rate, L is the core length, ΔP_f_ is the pressure drop across the core during the foam flooding, and ΔP_g_ is the pressure drop across the core during gas flooding. MRF is calculated by measuring and comparing the pressure differential along the core, during flooding by surfactant, or nanofluid-assisted foam flooding, or no foam condition (brine or gas flooding) at the same injection rate. The efficiency of the foam is higher at higher MRF, due to the control of mobility and early breakthrough.

Different types of nanoparticles such as SiO_2_, Al_2_O_3_, TiO_2_, CuO, and Fe_3_O_4_ have been used to develop nanofluid-based foams^31^. In most cases, nanoparticles are added to the surfactant at critical micelle concentration (CMC) to optimize the process. Gu et al.^32^ investigated the performance of foams generated by a surfactant at CMC with different concentrations of nanosilica. They also observed the effect of temperature and pressure alteration on the performances of the CMC-surfactant and nanofluid-assisted foams. According to their results, it was evident that an increase in temperature from 20 to 80 °C significantly decreased the foam average half-life by about 50%. Moreover, by comparing the foam stability based on increasing the NP concentration at the same temperatures, it was observed that increasing the NP concentration improves the half-life of the foam. Furthermore, they observed that by increasing the pressure from 2 to 8 MPa, the average half-life of the nanofluid-assisted foam increased by about 30%. Subsequently, their results demonstrated that the presence of the nanosilica (1 wt% SDS with 1 wt% nanosilica) enhanced the foam stability by improving the foam half-life by 34.3% in comparison to the standalone 1 wt% SDS (at 20 °C). They also ameliorated the channeling problem by improving the pressure drop by 21.1%, which shows an enhancement in MRF.

Sun et al.^33^ conducted a similar experimental study to investigate the effect of nanosilica at different concentrations. According to their results, the mixture of the surfactant and nanosilica improved the half-life by 97% and reduced the mobility by 50% compared to the standalone surfactant-based foam flooding. Lv et al.^34^ used different concentrations (0.3, 0.6, and 0.9 wt%) of nanosilica at CMC of sodium α-olefin sulfonate for the foam flooding experiment. The results demonstrated 54% improvement in MRF, which controlled the channeling, which was observed during the surfactant-based foam flooding. Ibrahim and Nasr-El-Din^35^ observed the effect of nanosilica with sodium α-olefin sulfonate in the presence and absence of the oil at two different temperatures of 77 and 150 °F. According to their findings, oil droplets have a detrimental effect on the effectiveness of the CMC-surfactant foam. Oil tends to spread across the interface of the foams, leading to a reduction in the stability of the lamellas. Consequently, it makes the foam decay faster by accelerating the foam drainage. Application of NP, even in the presence of the oil, increased the half-life. Nanosilica enhanced the foam MRF by about 23% in comparison to the surfactant-based foam. Ramanathan et al.^30^ studied the effect of the nanosilica and AOS foam flooding at the temperature of the 150 °F. Results demonstrated that nanosilica improved the half-life by 65% in comparison to the AOS foam. In addition, the MRF of the AOS foam was improved by about 17% after adding nanosilica. Sun et al.^36^ compared the half-life of the SDS and nanosilica at different temperatures of 20, 40, 50, 60, 70, and 80 °C. Results showed that nanosilica dramatically increased the stability of the foam in comparison to the SDS foam by 83, 88, 90, 91, 93, and 96% at each temperature, respectively. The presence of nanosilica improved the pressure differentials by 83%, preventing injection fluid channeling. Babamahmoudi and Riahi^37^ compared stability of the nanosilica foam and SDS foam in the absence and presence of the oil. They reported that oil significantly decreased the stability of the SDS foam. Nanosilica increased the stability of the SDS foam in the absence of the oil by about 23%. Wu et al.^38^ studied the effect of the nanosilica on the SDS foam stability with deionized water at 25 °C and 45 °C. They reported that the half-life of the nanosilica foam improved by about 5% in comparison to the SDS foam at 25 °C in the presence of the oil. Bayat et al.^39^ compared the effect of different concentrations of SiO_2_, Al_2_O_3_, TiO_2_, and CuO on CO_2_-foam stability. They reported that the SiO_2_ nanoparticles enhanced the half-life of the foam more than the other nanoparticles. Shi et al.^40^ investigated the effect of temperature (10–80 °C) and salinity (0–80,000 ppm) on the stability of the nanosilica foam. Despite the negative impact of increasing the temperature and salinity, nanosilica foam half-life enhanced by 51% in comparison to the base foam at 60 °C.

Based on the results reported in the literature, nanosilica is the best option to improve the properties of foams. Most of the studies have been conducted at CMC value of surfactant, which is reasonable to optimize the process. The objective of this study is to develop a simple model to estimate the effect of nanosilica concentrations on foam properties. Despite numerous studies conducted on the impact of NPs on the foam half-life and MRF, the optimum NP concentration range is not recommended, regardless of experimental conditions such as temperature, pressure, core permeability, and salinity. In this study, after development of a model, we conducted experimental studies to confirm the model.

## Methodology

The research methodology employed in this study aimed to investigate the potential of the combination of nanosilica and surfactant to enhance foam stability, viscosity, and reduce mobility. The assessment of foam properties and its application in the oil recovery process was conducted through foam stability, foam viscosity, and core flooding experiments.

Generally, for all the three experiments, sandstone cores, sodium dodecyl sulfate (SDS) surfactant powder, and silica nanoparticles were utilized. Brine was prepared in distilled water with sodium chloride.

**Figure 1:**
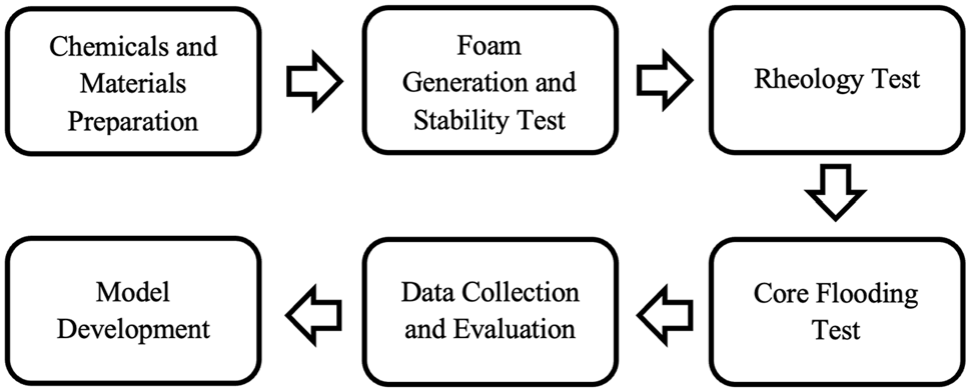
Flow diagram of experimental design.

Once all the nanofluid-surfactant solutions were prepared, foam half-life measurements were taken by the stability test for each concentration of nanoparticle. Followed by stability test, the rheological properties of the nanofluid-surfactant stabilized solutions were examined. In particular, the focus was made on the viscosity of the nanofluid-surfactant stabilized foam. Finally, core flooding test was carried out to evaluate the mobility reduction factor and general behavior of the solution in the porous media during gas injection. The flow for experimental design can be depicted in Fig. 1. A brief methodology for the laboratory tests is provided in the following section.

### Chemicals and materials

Nitrogen gas (N_2_), sodium chloride (NaCl), and sodium dodecyl sulfate (SDS) surfactant powder were provided by Sigma Aldrich; silica nanoparticle was purchased from Glantreo Limited Ireland as a dispersion of 25 wt%. Water-wet sandstone cores from the Bentheimer field were used. The cores were mostly composed of quartz (92%). The samples' properties are shown in Table 1. The porosity and permeability of the core samples were measured using Helium Porosimeter and Poroperm (from Vinci). This porous medium was selected due to the high mobility of the gas flow.

**Table 1:** Properties of core samples.

Core no	Diameter (mm)	Length (mm)	Porosity (%)	Permeability (mD)
1	37.87 ± 0.02	74.61 ± 0.02	22.63	2510
2	37.96 ± 0.02	74.56 ± 0.02	21.32	2430

### Experimental equipment

The foam stability test was conducted to evaluate the effect of nanosilica on surfactant-based foam, with a focus on measuring its half-life. The SRP 350 Steady State Relative Permeameter, manufactured by Vinci Technologies, was utilized for this purpose. The equipment configuration is illustrated in Fig. 1.

**Figure 2:**
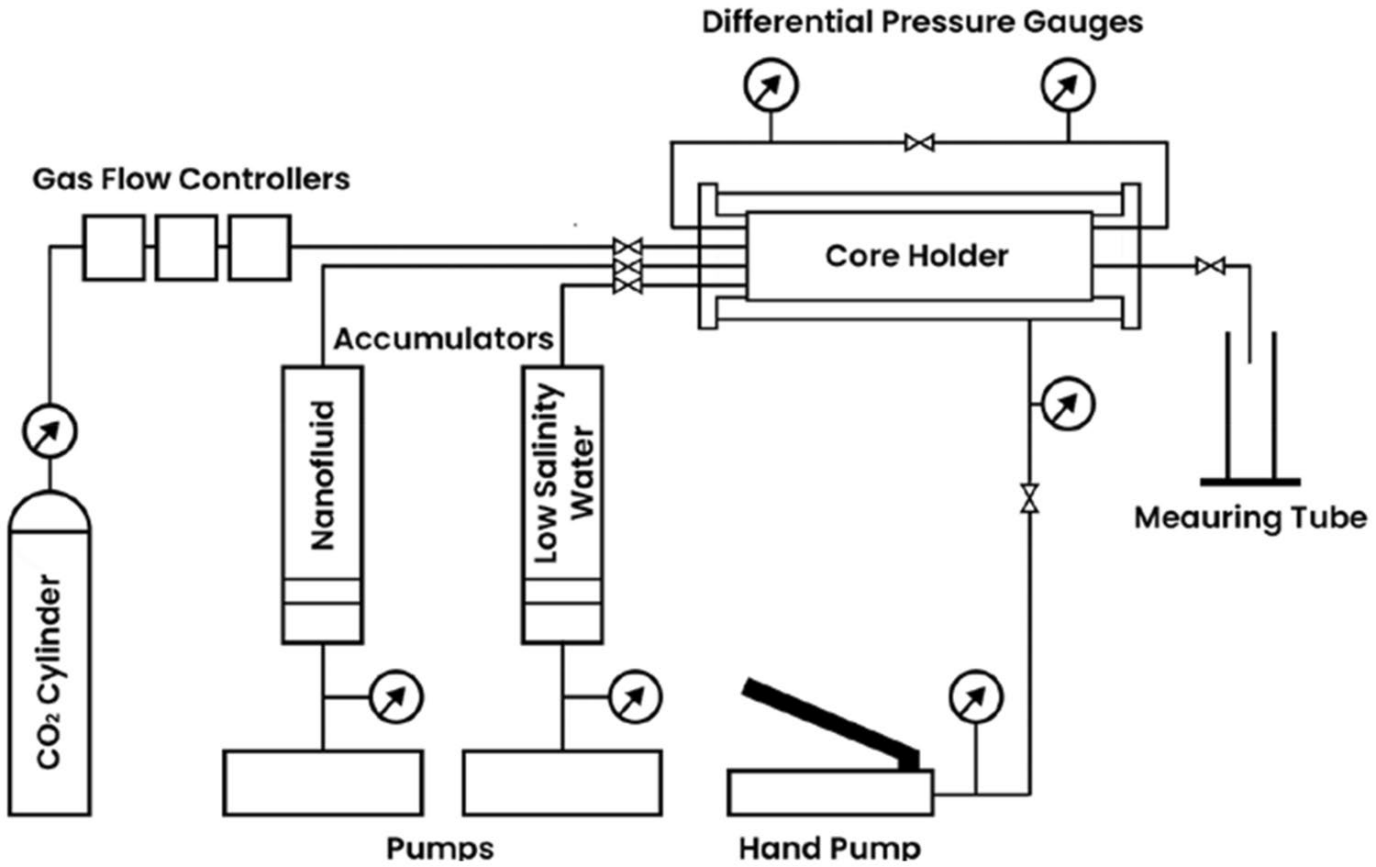
Steady State Relative Permeameter (SRP 350).

**Figure 3:**
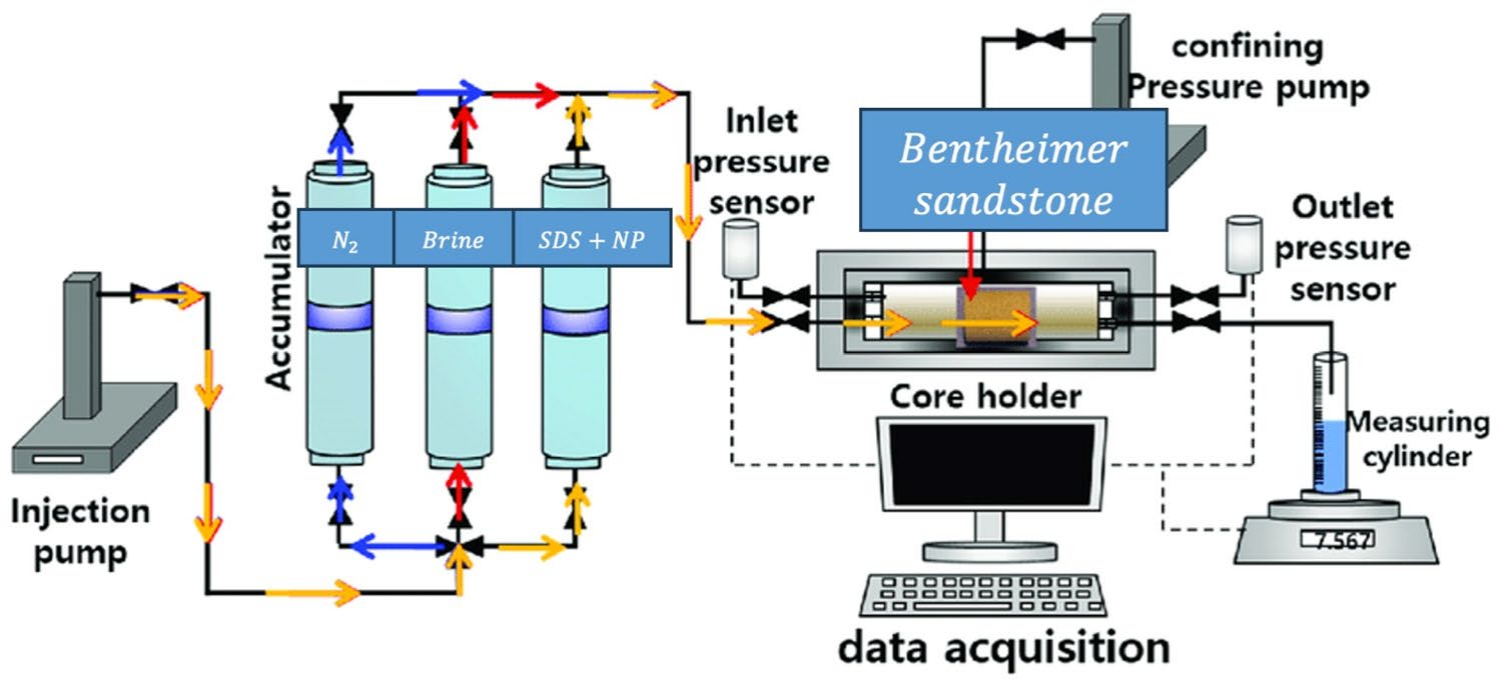
Core Flooding System (CFS 700).

To conduct the core flooding experiment, core plugs were saturated with formation brine utilizing the manual saturator. Subsequently, the foam flooding test was conducted using a core flooding system (CFS 700) developed by Vinci Technologies. The experimental setup is schematically depicted in Fig. 2. As shown in Fig. 3, the core flooding system consisted of an injection or syringe pump, three accumulators (accumulator A was filled with nitrogen gas, accumulator B was filled with brine solution, while accumulator C contained nanofluid-surfactant solution), a core holder, and a measuring cylinder. The inflow line, originating from the accumulators, was connected to the core holder containing a core sample.

### Experimental procedure

#### Foam stability test

For the foam stability test, overall, 5 samples with different nanosilica concentrations of 0.1, 0.2, 0.4, 0.6, and 0.8 wt% were used. The solution was prepared by adding 0.4 wt% of SDS surfactant to 10,000 ppm saline brine while mixing at 200–250 rpm on a magnetic stirrer. After 40 min of stirring, nanosilica particles were added to the solution and stirred for an additional 40 min. To ensure a homogeneous and stable dispersion of nanoparticles, as well as even distribution of surfactant molecules, the solution was placed into ultrasonic homogenizer for 90 min. Moreover, to ensure the stability of the prepared solution, it was physically observed for additional 35 min for any formation of precipitations. The solution was clear, no precipitation was formed which indicated the stability of the nanofluid-surfactant stabilized solution. After the preparation step, using the SRP 350, nitrogen gas was supplied into the flask with the nanosilica and surfactant mixture (100 ml for each of the mixture) for 5 min for foam generation until its level increased for an additional 50 ${{\text{cm}}}^{3}$. After that, half-life was measured by counting the time required to lose 50% of the original volume of the foam (25 ${{\text{cm}}}^{3}$).

#### Rheology test

For the rheology test, a total of 6 samples with different nanosilica concentrations of 0, 0.1, 0.2, 0.4, 0.6, and 0.8 wt% were prepared. The solutions were prepared similar to the samples used in the foam stability test. Subsequently, the solution of each of the concentrations was placed onto the rheometer plates. The rotational mode was used for the viscosity measurement of non-Newtonian fluid, in which the lower plate of the rheometer was rotated, and the upper plate was stationary. All the measurements were taken under ambient temperature conditions. The apparent viscosity value for each concentration of nanosilica at a constant shear rate of 10 1/s was reported.

#### Core flooding test

For each flooding test, a core sample was cleaned and dried. The core was subsequently brine-saturated with 10,000 ppm brine using manual saturator by applying 1100 psi pressure for 24 h. The core No. 1 was used for the surfactant-based foam flooding, while the core No. 2 was utilized for the nanofluid-based foam injection. As the primary stage of foam flooding, the fully saturated core sample was installed in the CFS 700 system into the core holder. The core was subjected to brine flooding at 3 different flow rates of 2 cc/min, 5 cc/min, and 8 cc/min to ensure complete saturation with the formation brine. The formation brine contained the same ratio of NaCl (10,000 ppm) in conformity with the critical salt concentration (CSC) to ensure that the risks associated with fines migration during oil recovery operations are minimized.

The first test included 5 alternating cycles of gas and surfactant-stabilized fluid injection. In particular, 3 cycles for ${{\text{N}}}_{2}$, and 2 cycles for SDS. While for the second flooding test, there were 13 alternating cycles of gas and nano silica- surfactant-stabilized fluid injection, 7 cycles for ${{\text{N}}}_{2}$ and 2 cycles for each nano silica concentration. A pressure of 300 psi was applied during all tests. All tests were conducted at a room temperature of 25 °C to avoid any effect of NPs thermal motion and at a flow rate of 2 cc/min. For each test, 3–4 pore volumes of solution were injected. Pressure drops at each stage were measured and foam production was monitored. As the final stage, the pressure drop data was measured to calculate MRF.

### Model development: collected data

In order to comprehensively analyze the effect of different concentrations of nanosilica on the foam stability and MRF, experimental data from various published sources were collected. The study considered key parameters, including core permeability, surfactant type at CMC, and varying concentrations of nanosilica. This approach ensures a comprehensive analysis of the relationship between nanosilica concentration and the performance of the foam in terms of stability and MRF. The data are given in Tables 2 and 3.

**Table 2:** Collected laboratory data of MRF for nanosilica and surfactants at CMC.

References	Permeability (mD)	Surfactant	NP Concentration (wt%)	MRF
Sun et al.^36^	851.1	SDS	2	6
	851.1	SDS	1	4
	851.1	SDS	0.5	3
	851.1	SDS	0.1	1.2
Rahmani^41^	60	NA	1	1.493
	45	NA	1	1.19
	25	NA	0.1	1.082
	10	NA	0.1	1.017
Ramanathan et al.^30^	113	AOS	0	2.9
	113	AOS	0.5	3.5
Gu et al.^32^	1121.5	SDS	1	2.538
	1217.2	SDS	1	2.284
	1226.56	SDS	0.5	2.284
	1163	SDS	0	2.086
Sun et al.^33^	2533	SDS	1	2.4
	2533	SDS	1	1.6
	2533	SDS	1	1.2
	2533	SDS	0	1.2
Lv et al.^34^	2533.15	AOS	0.9	11
	2261.5	AOS	0.75	6
	2464.22	AOS	0.6	9
	382	AOS	0.5	10.73
	382	AOS	0.3	8.72
	2261.57	AOS	0.3	6
	382	AOS	0.1	6.87
	382	AOS	0	4.85
	2388.23	AOS	0	5
Ibrahim and Nasr-El-Din^35^	111	AOS	0.1	1.3
	111	AOS	0	1

**Table 3:** Collected laboratory data of half-life for nanosilica with CMC- surfactant.

No	References	Surfactant	NP Concentration (wt%)	Half- life time (s)
1	Shi et al.^40^	SDS	3	18,000
2		SDS	1	18,000
9		SDS	0.5	18,000
11		SDS	0.3	13,500
15		SDS	0.1	9000
24		SDS	0.05	7500
44		SDS	0	1050
3	Sun et al.^33^	SDS	1	27,000
4		SDS	1	1200
5		SDS	1	2400
6		SDS	1	6000
7		SDS	1	12,000
32		SDS	0	600
8	Manan et al.^42^	AOS	1	2400
10		AOS	0.5	2520
13		AOS	0.3	2700
22		AOS	0.1	2760
45		AOS	0	1980
12	Wu et al.^38^	SDS	0.3	25,800
14		SDS	0.2	25,200
21		SDS	0.1	24,000
27		SDS	0.03	24,600
30		SDS	0.01	24,000
16	Bayat et al.^39^	NA	0.1	1080
25		NA	0.05	1380
26		NA	0.03	1740
28		NA	0.02	1620
29		NA	0.01	1680
17	Emrani et al.^28^	AOS	0.1	13,000
46		AOS	0	3000
18	Babamahmoudi and Riahi^37^	SDS	0.1	18,300
19		SDS	0.1	23,100
20		SDS	0.1	24,050
23	Dehdari et al.^43^	AOS	0.06	4020
31	Ibrahim and Nasr-El-Din^35^	AOS	0	900
33	Xu et al.^44^	AOS	0	165
34		AOS	0	220
35		AOS	0	235
36		AOS	0	250
37		AOS	0	260
38		AOS	0	270
39		AOS	0	280
40	Chen and Zhao^45^	AOS	0	420
41		AOS	0	450
42		AOS	0	450
43	Telmadarreie and Trivedi^46^	AOS	0	360

## Results and discussion

### Experimental measurement of foam parameters

#### Foam half-life

The foam half-life plays a pivotal role in assessing the stability of the foam and its potential for improving oil recovery in the foam flooding method. A longer half-life, corresponds to enhancing the stability of the foam, which implies a slower foam drainage rate. The experiment evaluated the effect of nanosilica on half-life of the foam under normal pressure, temperature, and 10,000 ppm salinity. The results presented in Table 4 demonstrate a significant increase in foam half-life with the addition of nanosilica to the CMC-surfactant foam. A comparison between the CMC-surfactant foam and the addition of 0.6 wt% nanosilica at CMC-surfactant indicates that NPs dramatically increase the half-life of the foam by 95.2%, which highlights the substantial effect of nanoparticles on the foam stability. A comparison between the half-life results of 0.6 and 0.8 wt% of NP reveal a 2.4% reduction, which highlights the importance of the optimum concentration of NP with CMC-surfactant. According to the results, it is apparent that the concentration range of NP between 0.4 and 0.6 wt% exhibits a significant improvement in the half-life of the foam.

**Table 4:** The experimental results of foam stability.

Type of foam	Composition of foam	Half-life (s)	Improvement rate (%)
SDS foam	0.4 wt% SDS	60	-
SDS foam with nanoparticles	0.4 wt% SDS + 0.1 wt% NP	150	60
	0.4 wt% SDS + 0.2 wt% NP	180	66.6
	0.4 wt% SDS + 0.4 wt% NP	360	83.3
	0.4 wt% SDS + 0.6 wt% NP	1260	95.2
	0.4 wt% SDS + 0.8 wt% NP	840	92.8

**Figure 4:**
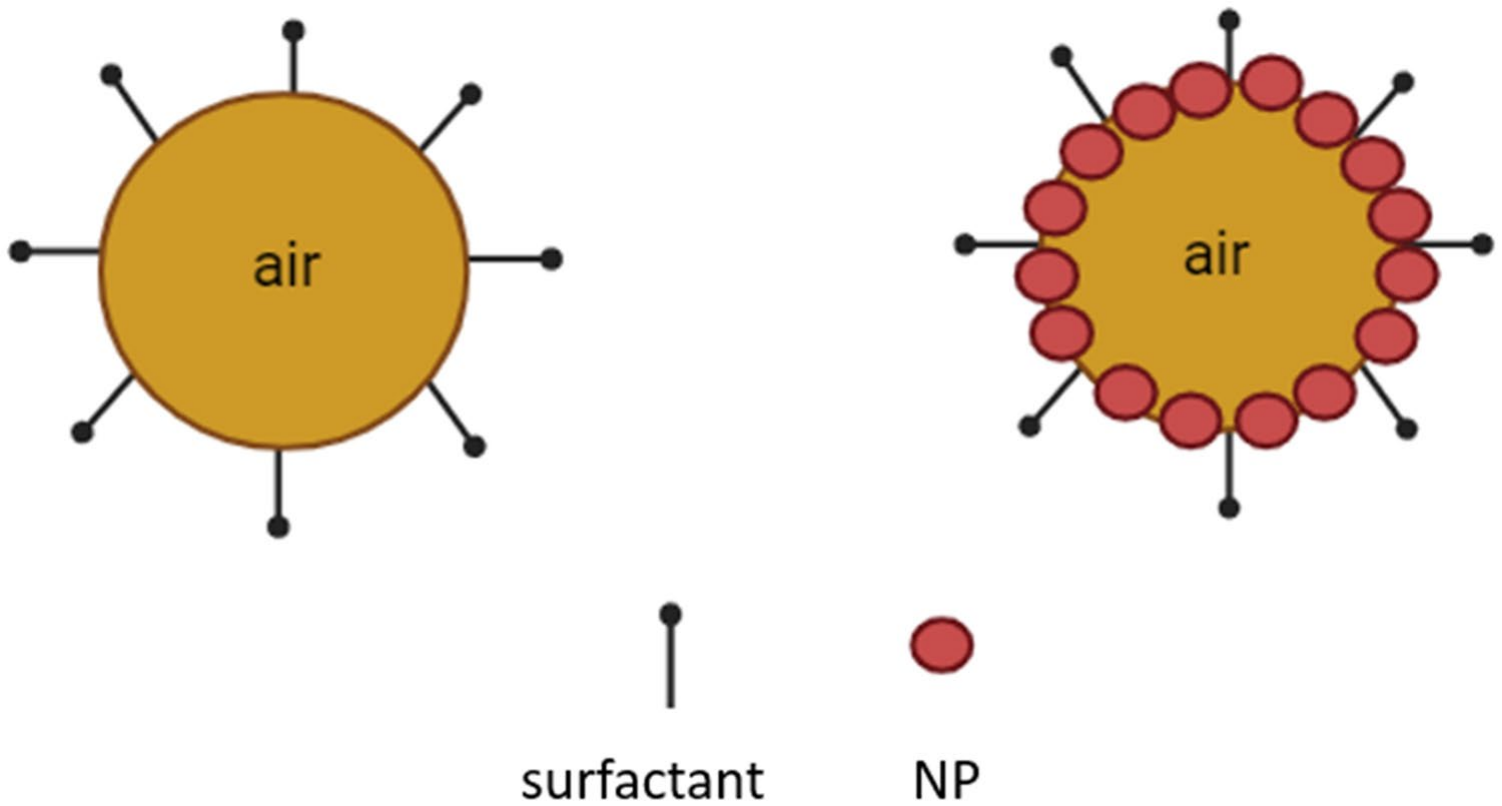
Schematic of foam surface stabilized by NP with CMC-surfactant.

Adding an optimal concentration range of NP to the CMC-surfactant increases the total concentration of the foam, which significantly densifies the foam. Moreover, the presence of NP in the foam contributes to a reduction in interfacial tension (IFT) by promoting the tension between foam lamella and solid particles. This improvement can be attributed to the fact that the NP forms a layer on the surface of the foam (Fig. 4), which effectively reduces the interface of the liquid/ gas phase and blocks the pore channels. By reducing the interface between the liquid and gas phases, as well as blocking pore channels, the drainage pathways for the liquid phase become more restricted. The combination of reduced IFT and blocked pore channels results in a more stable foam system. The slower drainage rate allows the foam to persist for a longer duration without collapsing.

**Figure 5:**
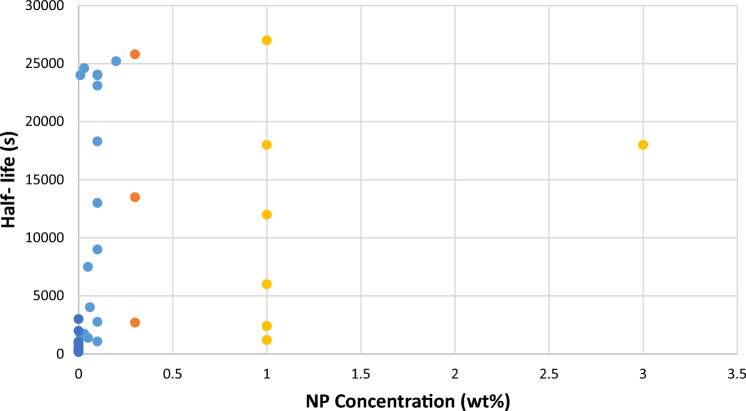
Comparing collected laboratory data of half-life for nanosilica with CMC-surfactant foam.

Additionally, several other parameters should be considered experimentally. Referring to Fig. 5, the zero concentration shows the base case, when only surfactant is present in the foam at CMC. As can be observed from these data, different measurements are reported at any NP concentration, due to the difference in temperature, pressure, and salinity. These parameters affect the adsorption of the surfactant on the surface of the foams. For example, increasing the pressure positively affects the foam half-life. Higher pressure densifies the foam structure making the foam more stable. Furthermore, increasing the salinity results in the higher adsorption of surfactant on the foam's surface, higher surface tension, stronger lamellae, and higher apparent viscosity. On the other hand, temperature exhibits a detrimental effect on the foam stability. However, the presence of NPs results in greater strength against high thermal conditions in comparison to the standalone CMC-surfactant foams.

**Figure 6:**
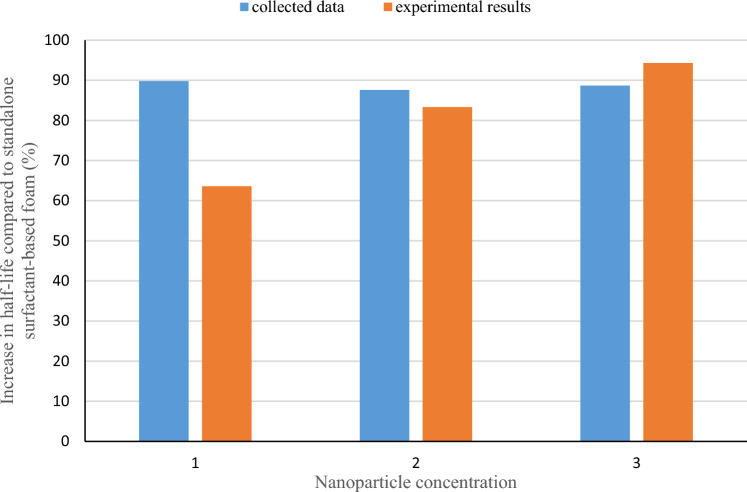
Comparison of the average improvement rate of collected half-life and experimental result (1: less than 0.4; 2: 0.4–0.6; and 3: more than 0.6).

Analysis of the collected data demonstrates that higher NP concentrations, at the same temperature/salinity/pressure conditions, results in an improvement in the half-life of the foam. The variation in half-life observed in Fig. 5 is attributed to different temperature, pressure, and salinity conditions. So, to study the improvement of the half-life, as a rule of thumb, an average number was calculated for each range of NP, based on the available data in the literature, as shown in Fig. 6. The incorporation of NP concentrations between 0–0.2, 0.2–0.4, 0.4–0.6, and more than 0.6 wt% to the CMC-surfactant foam resulted in substantial improvement in the average half-life of the foams by 90%, 88.4%, 87.6%, and 88.7%, respectively. Hence, data in the literature shows an improvement of about 90%, which agrees with what we observed in our experiments. Our measurements showed that the concentration of 0.4–0.6% shows better results and can be considered as an optimum condition.

#### Foam viscosity

The viscosity of the foam has a critical role in enhancing the displacement mobility ratio in foam flooding processes. In this study, a weak foam was generated by using SDS surfactant. By adding 0.4, 0.6, and 0.8 wt% nanosilica to the CMC-surfactant solution, the viscosity of the foam was improved by 56.1%, 81.6%, and 1.25%, respectively. The data presented in Table 5 clearly demonstrates that increasing the concentration of NP in the CMC-surfactant foam leads to an improvement in the strength of the foam by increasing the foam viscosity in comparison to the CMC-surfactant alone. By increasing the viscosity of the foam, the density of the foam is also increased, which leads to improved foam stability. In addition, an increase in the viscosity of the foam helps to plug high permeability pores. Plugging the pores mitigates the water channeling by optimizing the water injection process. This phenomenon improves the efficiency of the displacement fluid by improving the MRF.

**Table 5:** The experimental results of foam viscosity.

Type of foam	Composition of foam	Apparent viscosity at shear rate 10 (mPa s)
SDS foam	0.4 wt% SDS	0.79
SDS foam with nanoparticles	0.4 wt% SDS + 0.4 wt% NP	1.8
	0.4 wt% SDS + 0.6 wt% NP	4.3
	0.4 wt% SDS + 0.8 wt% NP	0.8

Moreover, it is important to highlight that based on the experimental results, the optimal NP concentration range is observed to be between 0.4 and 0.6 wt%, due to the substantial improvement in the apparent viscosity of the foam. However, increasing the NP concentration above the optimal range can lead to a reduction in the viscosity of the foam due to increasing the surface tension gradient and NP aggregate.

#### Mobility reduction factor

**Figure 7:**
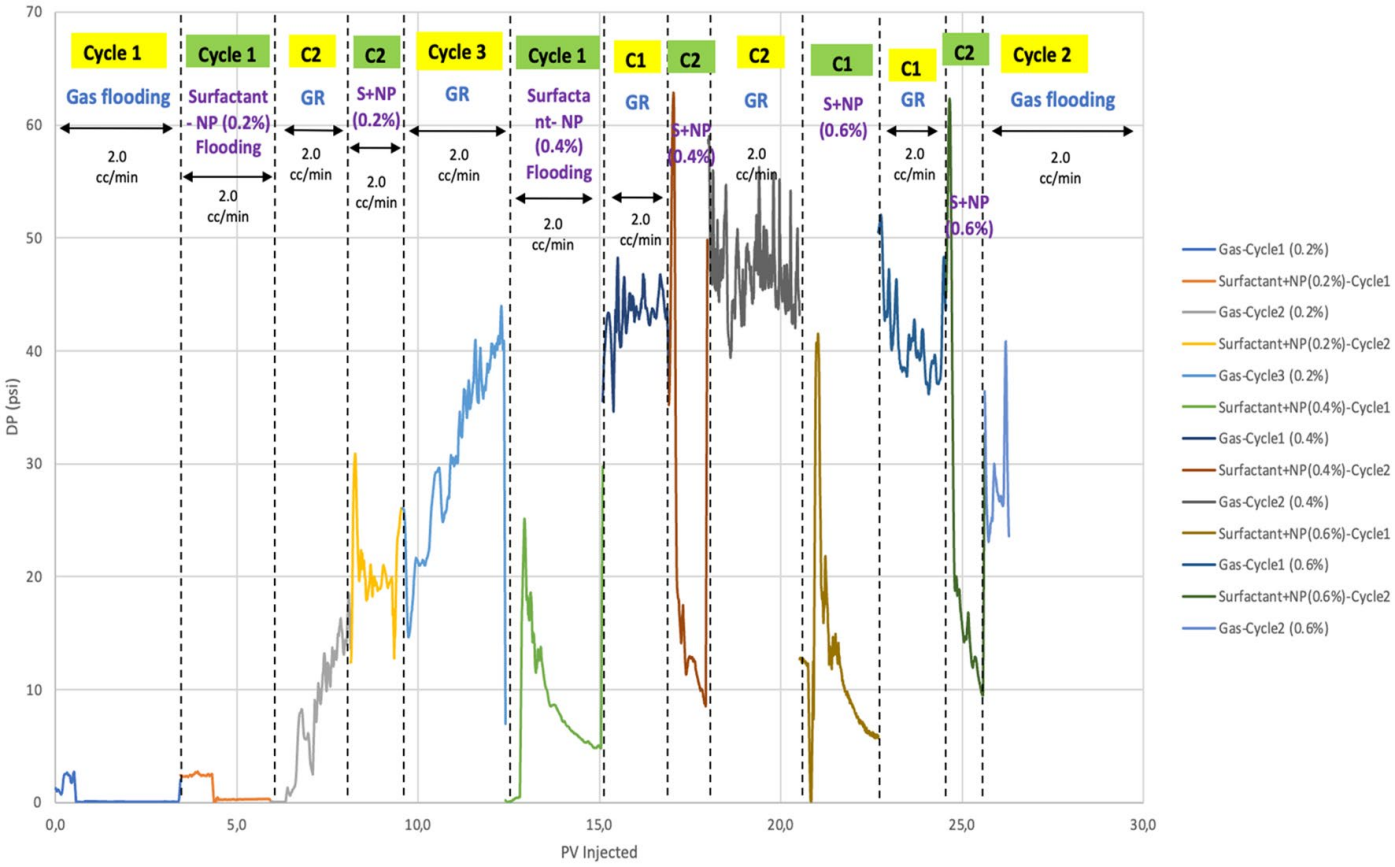
Pressure changes during the core flooding experiment.

Mobility control plays a significant role in preventing viscous fingering and gravity segregation. By incorporating 0.2, 0.4, and 0.6 wt% of nanosilica into the CMC-surfactant, nanoparticle emulsion is stabilized thereby improving the mobility reduction factor. Figure 7 shows pressure drop during different steps of nanofluid-assisted foam flooding. An increase in pressure drop was observed at higher NP concentrations. By comparing pressure drop values, MRF was calculated and shown in Table 6. It is evident that a 0.2 wt% concentration of nanosilica combined with the CMC-surfactant improves the MRF of the foam by 43.1%. Moreover, increasing the NP concentration to 0.4 and 0.6 wt% results in a significant improvement in the MRF by 92.06% and 94.9%, respectively. It is apparent that the optimal concentration range for NP, in order to improve the mobility ratio, is between 0.4 and 0.6 wt%. A comprehensive comparison of the optimum concentration range for both results of half-life and MRF, shows that the best concentration is 0.4–0.6 wt%. Lower concentrations of NP do not generate a strong foam that control the mobility.

**Table 6:** Measured data of Mobility reduction factor.

Type of foam	Composition of foam	MRF
SDS foam	0.4 wt% SDS	0.62
SDS foam with nanoparticles	0.4 wt% SDS + 0.4 wt% NP	1.09
	0.4 wt% SDS + 0.6 wt% NP	7.81
	0.4 wt% SDS + 0.8 wt% NP	12.21

**Figure 8:**
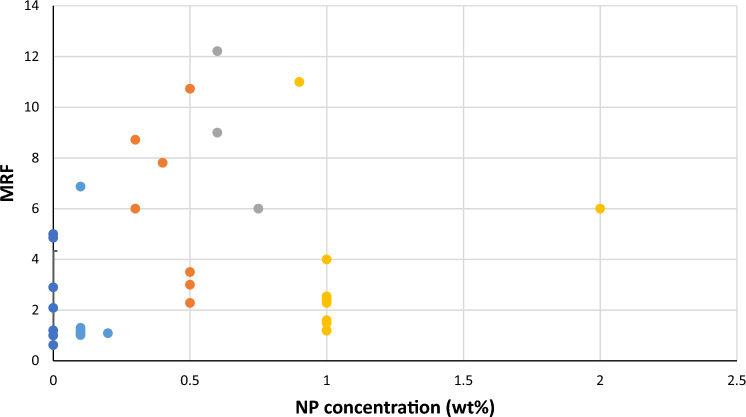
Comparing collected laboratory data of MRF for nanosilica with CMC-surfactant foam.

**Figure 9:**
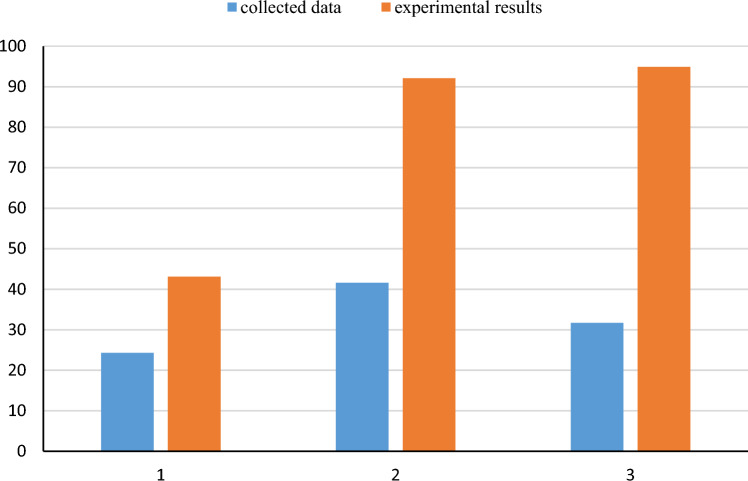
Comparison of the average improvement rate of collected MRF and experimental result (1: 0–0.4; 2: 0.4–0.6; 3: more than 0.6 wt%).

It should be noted that the application of very low concentrations of NP (less than 0.4 wt%) does not have a significant effect on MRF due to the generation of weak foams. Figures 8 and 9 demonstrates that the concentration range of 0.4–0.6 wt% exhibits a significant improvement in the MRF, with an enhancement of about 90%. Higher concentration does not show more improvement in the performance of the foam. So, by comparing the average increase rate derived from collected data and the average improvement of the MRF of our experimental results, it is evident that there is an alignment between the optimal concentration ranges of NP between the two data series. MRF and half-life data showed that the range of 0.4–0.6 wt% is the optimum option for the design of nanofluid-based foam. Concentrations lower than this number are weak, and higher than this range does not show noticeable improvement, as NPs have occupied surfaces on the foam droplets.

## Conclusion

The study aims to investigate the advantageous impact of nanosilica when combined with the CMC- surfactant on foam stability, apparent viscosity, and mobility reduction factor (MRF) based on different ranges of NP concentration.

Based on the experimental results, it has been determined that the optimal concentration range of nano silica when combined with CMC-surfactant is 0.4–0.6 wt%. According to the collected data, in the given range, the use of NP dramatically increases the half-life by approximately 90%, making foam more stable. In addition, nanofluid-based foams reduce the mobility ratio by about 20–40% more than standalone surfactant-based foams, which controls macroscopic sweep efficiency-related problems such as fingering and override.

## Data Availability

The data that support the findings of this study are available from the corresponding author, upon reasonable request.

## References

[1] Lake, L.W., Schmidt, R. L., & Venuto, P. B. A niche for enhanced oil recovery in the 1990s. Oilfield Review (The Netherlands). **4**(1) (1992).

[2] Orr FM, Heller JP, Taber JJ (1982). Carbon dioxide flooding for enhanced oil recovery: Promise and problems. J. Am. Oil Chem. Soc..

[3] Hoffman BT, Shoaib S (2013). CO2 flooding to increase recovery for unconventional liquids-rich reservoirs. J. Energy Resour. Technol..

[4] Li D (2015). CO2-sensitive foams for mobility control and channeling blocking in enhanced WAG process. Chem. Eng. Res. Des..

[5] Lee S, Kam S (2013). Enhanced oil recovery by using CO2 foams: fundamentals and field applications. Enhanced Oil Recovery Field Case Studies.

[6] Rossen WR (2017). Foams in enhanced oil recovery. Foams.

[7] Heller J (1985). Direct thickeners for mobility control of CO2 floods. Soc. Pet. Eng. J..

[8] Martin DF, Taber J (1992). Carbon dioxide flooding. J. Petrol. Technol..

[9] Kovscek AR, Radke CJ (1994). Fundamentals of Foam Transport in Porous Media. Foams: Fundamentals and Applications in the Petroleum Industry.

[10] Worthen, A.J., et al. *Nanoparticle stabilized carbon dioxide in water foams for enhanced oil recovery*. in *SPE Improved Oil Recovery Symposium*. 2012. OnePetro.

[11] Koval E (1963). A method for predicting the performance of unstable miscible displacement in heterogeneous media. Soc. Petrol. Eng. J..

[12] Kuhlman M (1992). CO2 foam with surfactants used below their critical micelle concentrations. SPE Reserv. Eng..

[13] Chang Y-B (1994). CO2 flow patterns under multiphase flow: heterogeneous field-scale conditions. SPE Reserv. Eng..

[14] Yaghoobi, H., & Heller, J. Laboratory investigation of parameters affecting co2-foam mobility in sandstone at reservoir conditions. in *SPE Eastern Regional Meeting* (SPE, 1994).

[15] Khalil F, Asghari K (2006). Application of CO2-foam as a means of reducing carbon dioxide mobility. J. Can. Pet. Technol..

[16] Waggoner J, Castillo J, Lake LW (1992). Simulation of EOR processes in stochastically generated permeable media. SPE Form. Eval..

[17] Yekeen N (2017). Influence of surfactant and electrolyte concentrations on surfactant Adsorption and foaming characteristics. J. Petrol. Sci. Eng..

[18] Schramm, L.L., *Foams: fundamentals and applications in the petroleum industry* (ACS Publications, 1994).

[19] Chou, S., *et al*. CO2 foam field trial at north ward-estes. In *SPE annual technical conference and exhibition*. OnePetro (1992).

[20] Blaker T (2002). Foam for gas mobility control in the Snorre field: The FAWAG project. SPE Reservoir Eval. Eng..

[21] Singh R, Mohanty KK (2016). Foams stabilized by in-situ surface-activated nanoparticles in bulk and porous media. SPE J..

[22] Talebian, S. H., *et al.**Foam assisted CO2-EOR; concepts, challenges and applications*. in *SPE Asia Pacific Enhanced Oil Recovery Conference*. 2013. SPE.

[23] Falls A (1988). Development of a mechanistic foam simulator: The population balance and generation by snap-off. SPE Reserv. Eng..

[24] Guo H (2012). A novel alkaline/surfactant/foam enhanced oil recovery process. SPE J..

[25] Tang J, Ansari MN, Rossen WR (2019). Quantitative modeling of the effect of oil on foam for enhanced oil recovery. SPE J..

[26] Zhou Z, Rossen W (1995). Applying fractional-flow theory to foam processes at the "limiting capillary pressure". SPE Adv. Technol. Ser..

[27] Lee HO, Heller JP (1990). Laboratory measurements of CO2-foam mobility. SPE Reserv. Eng..

[28] Emrani, A. S., & Nasr-El-Din, H. A. *Stabilizing CO2-foam using nanoparticles*. in *SPE European Formation Damage Conference and Exhibition*. 2015. SPE.

[29] Khandoozi S (2023). A comparative analysis of the effect of nanoparticles/surfactant assisted foam injection on the gas mobility control at different heterogeneities. Fuel.

[30] Ramanathan R, Abdelwahab O, Nasr-El-Din HA (2021). A new effective multiwalled carbon nanotube-foam system for mobility control. SPE J..

[31] Kumar S, Mandal A (2017). Investigation on stabilization of CO2 foam by ionic and nonionic surfactants in presence of different additives for application in enhanced oil recovery. Appl. Surf. Sci..

[32] Gu Z (2022). Experimental investigation on the SiO2 nanoparticle foam system characteristics and its advantages in the heavy oil reservoir development. J. Pet. Sci. Eng..

[33] Sun Q (2015). Aqueous foam stabilized by partially hydrophobic nanoparticles in the presence of surfactant. Colloids Surf. A Physicochem. Eng. Aspects.

[34] Lv Q (2021). CO2 mobility control in porous media by using armored bubbles with silica nanoparticles. Ind. Eng. Chem. Res..

[35] Farid Ibrahim, A., & Nasr-El-Din, H. *Stability improvement of CO2 foam for enhanced oil recovery applications using nanoparticles and viscoelastic surfactants*. In *SPE Trinidad and Tobago Section Energy Resources Conference* (2018).

[36] Sun Q (2014). Utilization of surfactant-stabilized foam for enhanced oil recovery by adding nanoparticles. Energy Fuels.

[37] Babamahmoudi S, Riahi S (2018). Application of nano particle for enhancement of foam stability in the presence of crude oil: Experimental investigation. J. Mol. Liquids.

[38] Wu X (2023). Synergistic effects between anionic surfactant SDS and hydrophilic silica nanoparticles in improving foam performance for foam flooding. J. Mol. Liquids.

[39] Bayat AE, Rajaei K, Junin R (2016). Assessing the effects of nanoparticle type and concentration on the stability of CO2 foams and the performance in enhanced oil recovery. Colloids Surf. A Physicochem. Eng. Aspects.

[40] Shi G (2021). Visualized study of a nanoparticle-assisted foam system to enhance oil recovery by nuclear magnetic resonance online flooding experiment. Energy Fuels.

[41] Rahmani O (2018). Mobility control in carbon dioxide-enhanced oil recovery process using nanoparticle-stabilized foam for carbonate reservoirs. Colloids Surf. A Physicochem. Eng. Aspects.

[42] Manan MA (2015). Effects of nanoparticle types on carbon dioxide foam flooding in enhanced oil recovery. Pet. Sci. Technol..

[43] Dehdari B (2020). New insight into foam stability enhancement mechanism, using polyvinyl alcohol (PVA) and nanoparticles. J. Mol. Liquids.

[44] Xu X, Saeedi A, Liu K (2016). Experimental study on a novel foaming formula for CO2 foam flooding. J. Energy Resour. Technol..

[45] Chen Z, Zhao X (2015). Enhancing heavy-oil recovery by using middle carbon alcohol-enhanced waterflooding, surfactant flooding, and foam flooding. Energy Fuels.

[46] Telmadarreie, A., & Trivedi, J. J. *Static and Dynamic Performance of Wet Foam and Polymer-Enhanced Foam in the Presence of Heavy Oil*. Colloids and Interfaces, **2** (2018).

